# A Double-Layer Vehicle Speed Prediction Based on BPNN-LSTM for Off-Road Vehicles

**DOI:** 10.3390/s23146385

**Published:** 2023-07-13

**Authors:** Jichao Liu, Yanyan Liang, Zheng Chen, Huaiyi Li, Weikang Zhang, Junling Sun

**Affiliations:** 1 Jiangsu XCMG Research Institute Co., Ltd., Xuzhou 221004, China; liujichao@xcmg.com (J.L.); liangyanyan@xcmg.com (Y.L.); lihuaiyi@xcmg.com (H.L.); zhangweikang@xcmg.com (W.Z.); sunjunling@xcmg.com (J.S.); 2 School of Materials and Physics, China University of Mining and Technology, Xuzhou 221116, China

**Keywords:** vehicle speed prediction, BPNN, LSTM, non-road vehicles

## Abstract

The accurate prediction of vehicle speed is crucial for the energy management of vehicles. The existing vehicle speed prediction (VSP) methods mainly focus on road vehicles and rarely on off-road vehicles. In this paper, a double-layer VSP method based on backpropagation neural network (BPNN) and long short-term memory (LSTM) for off-road vehicles is proposed. First of all, considering the motion characteristics of off-road vehicles, the VSP problem is established and the relationship between the variables in the problem is carefully analyzed. Then, the double-layer VSP framework is presented, which consists of speed prediction and information update layers. The speed prediction layer established by using LSTM is to predict vehicle speed in the horizon, and the information update layer built by BPNN is to update the prediction information. Finally, with the help of mining truck and loader operation scenarios, the proposed VSP method is compared with the analytical method, BPNN prediction method, and recurrent neural network (RNN) prediction method in terms of speed prediction accuracy. The results show that, under the premise of ensuring the real-time prediction performance, the average prediction error of the proposed BPNN-LSTM prediction method under two operation scenarios reduces by 48.14%, 35.82% and 30.09% compared with the other three methods, respectively. The proposed speed prediction method provides a new solution for predicting the speed of off-road vehicles, effectively improving the speed prediction accuracy.

## 1. Introduction

Transportation plays an important role in social and economic development, which is the foundation of a country’s rapid and sustainable development. With the progress of society, it is difficult for traditional transportation technology to cope with the increasing traffic pressure [1]. The intelligent transportation system (ITS) emerges with the help of big data and artificial intelligence [2,3]. Accurate vehicle speed prediction (VSP) is not only an important component of ITS, but also the key to achieving energy optimization.

At present, in terms of on-road vehicles, the existing VSP methods are mainly divided into mechanism modeling and data-driven methods [4]. The mechanism modeling method is to predict the information of the target driving condition according to traffic flow theory, which mainly includes dynamic models, macro models, and micro models [5,6]. However, in the actual situation, the vehicle motion process is affected by its own motion state and external traffic state, which makes it difficult for the VSP model established by mechanism modeling method to effectively reflect the real motion process of the vehicle. Therefore, the data-driven VSP methods, such as the Markov model and artificial neural network (NN) model [7], have been proposed, which can predict the future speed of the vehicle based on the current and historical condition information [4,8]. In other words, the data-driven VSP method gives full play to the role of current and historical traffic data, and weakens the dependence on complex physical traffic models. For the Markov model, the dimension of the Markov probability transfer matrix (MPTM) will rapidly increase with the increase in the vehicle motion state, resulting in a larger calculation and limiting its vehicle speed prediction effect. For this purpose, the neural network speed prediction model represented by the backpropagation NN (BPNN), convolutional neural network (CNN), and recurrent NN (RNN) has been proposed. Although BPNN and CNN have a strong fitting ability for nonlinear systems, the continuity system between data will weaken their predictive ability. Furthermore, the increase in time series length will lead to the disappearance and explosion of the gradient of the RNN, limiting the speed prediction effect of the RNN. Therefore, with the advantage of information inheritance, the long short-term memory (LSTM) and gated recurrent unit (GRU) of RNN variants [1] have been widely used in speed prediction models evolving according to time series.

Recently, under the traction of the national dual-carbon policy, construction machinery has also moved towards the development of electrification. The driving conditions of new-energy construction machinery also need to be analyzed, especially for mine cluster operations. However, the exiting VSP methods mainly focus on road vehicles, rarely aiming at non-road vehicles. The successful application of the VSP method in road vehicles provides an effective solution for non-road vehicles to achieve energy conservation and emission reduction [9]. Under normal circumstances, the non-road vehicles mainly drive on roads with random structure and lack sufficient traffic information, making the speed prediction of non-road vehicles more difficult than road vehicles. Therefore, taking mine operation as a scenario, this paper makes full use of the working conditions of non-road vehicles and proposes a double-layer speed prediction method based on BPNN-LSTM for non-road vehicles. First of all, the vehicle motion state data is collected and analyzed to clarify the impact of slope and load on vehicle speed and acceleration. On this basis, we build the offline working condition database. Then, a road state prediction model is established by using a BPNN. Then, a vehicle speed prediction model based on BPNN-LSTM is proposed, containing speed prediction and information update layers. The speed prediction layer is built using LSTM to predict vehicle speed in the horizon, and the information update layer is established by the BPNN to update the prediction information. Finally, the speed prediction effect of the proposed method will be verified on the hardware in loop (HIL) platform.

Accordingly, the structure of this paper is as follows. The works related to VSP are reviewed in Section 2. Section 3 gives the prediction problem model and the analysis of working condition characteristics. The double-layer speed prediction method based on BPNN-LSTM is established in Section 4. The discussion of the simulation and results are shown in Section 5. The conclusion and future work are shown in Section 6.

## 2. Related Works

In order to realize energy optimization management, it is very important for the energy management strategy to accurately predict the information of future working conditions [10]. In other words, selecting a reasonable VSP method is critical to non-road vehicles. The existing VSP methods are mainly divided into mechanism modeling and data-driven methods.

Generally, the mechanism modeling of VSP is established according to the vehicle kinematics or motion relationship between vehicles. Specifically, Gong et al. [5] built a simple VSP model based on vehicle kinematics by analyzing vehicle acceleration, deceleration and maximum speed. Considering the influence of traffic flow on the motion state of individual vehicles, the VSP model based on traffic flow was subsequently proposed [11,12,13], and the traffic flow speed was regarded as the future speed of the vehicle. Morlock et al. [14] further used the collected traffic data to analyze the probability of stop modes of individual vehicles, and built the acceleration and deceleration models. However, for the individual vehicle, compared with macro traffic flow, the motion states of neighbor vehicles are more likely to affect its driving speed in the actual traffic situation, and the VSP model based on car-following was designed in [15,16]. The speed values of front vehicles were regarded as the future speed trajectory of the self-vehicle. By means of the advantages of the Internet of Things (IOT), Jisu et al. [17] detected the current position of the vehicle through roadside sensors, and estimated the velocity by using a Kalman filter. In order to further improve the speed prediction accuracy, the adaptive extended Kalman filter (AEKF) algorithm for VSP was designed in [18]. Contrasted with the conventional extended Kalman filter (EKF) algorithm, the AEKF algorithm improves the precision of the mean square error (MSE) and mean absolute error (MAE) by 57.4% and 32.4%, respectively.

Then, taking account of the randomness and nonlinearity of the vehicle motion state, various types of data-driven VSP models were presented with the help of the prediction ability of a probability transfer matrix and artificial NN. For example, considering that the change process of vehicle motion states has obvious Markov characteristics, the VSP model based on the Markov chain was presented in [19], estimating the future vehicle speed by constructing the MPTM of vehicle motion states. Ding et al. [20] assumed that the vehicle acceleration at each moment had nothing to do with historical information and was only determined by current information, and established a speed-acceleration MPTM to predict the short-term vehicle speed sequence. Jaewook et al. [21] utilized a Markov chain with speed constraints to design a speed prediction algorithm, achieving a 3.8041 km/h root-mean-square error (RMSE) with a prediction horizon of up to 200 m. Yang et al. [22] selected the vehicle speed, acceleration, jerk, road lane, traffic speed and volume to characterize the driver’s driving behavior, and designed an Oriented Hidden Semi-Markov Model (Oriented-HSMM) to learn and predict the driver’s driving preference sequences. Nevertheless, the dimension of the MPTM rapidly increase with the increase in the vehicle motion state, making the real-time calculation of the MPTM become worse and limiting its vehicle speed prediction effect.

For that, the VSP model based on an NN was eventually proposed. For ensuring the VSP accuracy, Ladan et al. [23] utilized the sliding window time series (SWTS) to determine the prediction window size, and proposed an evolutionary least learning machine (E-LLM) to predict the short-term vehicle speed sequence. In [24], a two-level prediction system based on an NN and hidden Markov model (HMM) for VSP was designed. Yan et al. [25] analyzed the influence of historical vehicle speed and its corresponding acceleration, steering information, location and driving date on the future speed, and established a VSP model based on a deep NN (DNN). Considering the impact of the driver–vehicle–road system on the actual speed profile, a VSP model was established by combining niche immune genetic algorithm-support vector machine (NIGA-SVM) and genetic algorithm-support vector machine (GA-SVM) prediction algorithms in [26], improving the accuracy and timeliness of vehicle speed forecasting. In view of the time-varying and nonlinear nature of vehicle speed, a CNN-based architecture with two-channel input was proposed for predicting short-term speed [27]. Katariya et al. [28] designed the temporal convolutional networks (TCNs) for VSP, which can provide more robust time prediction with less computation compared with traditional CNNs. In [1], to overcome the limitations of the single prediction method, a short-term traffic speed prediction model was presented by combining an improved TCN and graph convolution network (GCN). The time dimension and local spatial dimension features were extracted by the improved TCN, and the topological relationship between road nodes was extracted by GCN. Finally, both spatial and temporal features were combined with road parameters to achieve accurate short-term traffic speed prediction. In addition, the BPNN has also been used for VSP [29,30,31,32]. For instance, in [29], a long-term VSP model was designed by using a BPNN, which employed a genetic algorithm (GA) to optimize model parameters to improve the accuracy of speed prediction. Guo et al. [30] designed an adaptive particle swarm optimization–least squares support vector machine (APSO-LSSVM) for VSP, and utilized a BPNN to establish a local high-precision nonlinear fitting relationship between the predicted value and deviation, achieving a correction of the prediction value. Making full use of the advantages of Markov and the BPNN, Ref. [32] designed an extraction method suitable for fixed-route vehicle speed. The results show that the combined prediction model can improve the prediction accuracy by 25.3% on average compared with the Markov prediction model.

Additionally, the vehicle state changes continuously with time or space, i.e., the information between adjacent states is inherited. Therefore, the RNN, LSTM, and GRU, possessing the advantage of information inheritance, have been used for VSP up until the present [7,8,33,34]. In [7], the RNN was used to establish the VSP model for an ego-vehicle, whose prediction accuracy and execution time were compared with the Markov chain model. To predict the vehicle speed, Zhang et al. [33] built an inflated 3D inception LSTM network by combining the spatiotemporal vision information and vehicle motion states, achieving a high accuracy of speed prediction in various traffic densities. A freeway traffic speed prediction model based on a GRU was established in [8] to realize traffic flow speed prediction for freeways. Zafar et al. [34] used LSTM and a GRU to build a speed prediction model for a road segment, which outperforms the rest with an RMSE of 4.5 and mean absolute percentage error (MAPE) of 6.67%. Xu et al. [35] analyzed the speed prediction effect of a multi-layer perception NN (MLPs-NN), LSTM-NN and GRU-NN. The results show that the prediction ability of GRU-NN is more prominent.

Based on above analysis, the existing VSP methods mainly focus on road vehicles, rarely aiming at non-road vehicles. Moreover, the working conditions of non-road vehicles are more complex and changeable, with complex operating scenarios and strong periodicity. As a result, to overcome the shortcomings of the existing VSP methods, this paper proposes a double-layer speed prediction method based on BPNN-LSTM for non-road vehicles.

## 3. Methodology

### 3.1. Problem Description

**Figure 1:**
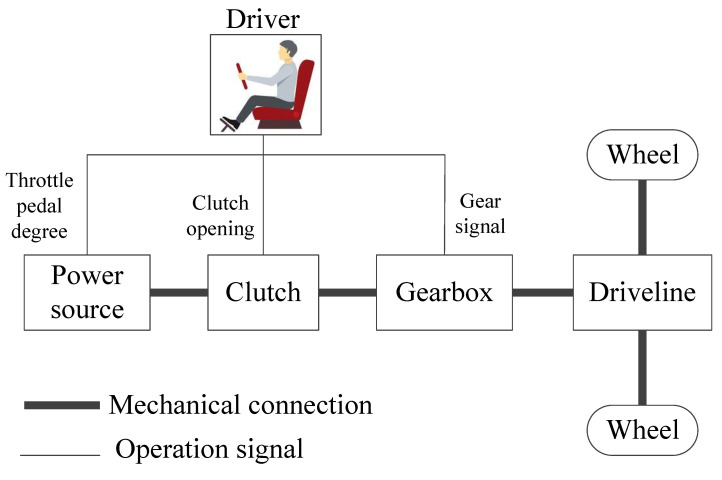
Powertrain topology of a vehicle.

The non-road vehicle generally works in closed working areas. Therefore, its driving state is mainly affected by the vehicle itself and the road structure. According to vehicle theory, when the vehicle is regarded as a particle, its motion state can be expressed by the following kinematics equation:

(1) $$\left\{\begin{array}{l} v=\dot{l}\\ a=\dot{v} \end{array}\right.$$

where l, v and a are denoted by the driving distance, vehicle speed and acceleration, respectively. Furthermore, when the vehicle is seen as an individual system, as shown in Figure 1, its state space equation in discrete time domain can be written as (assuming the time step is 1 (s))

(2) $$v(t+1)=v(t)+\frac{T_{\mathrm{req}}(t)}{r_{\mathrm{wh}}\cdot m}-\frac{C_{a}\cdot \rho_{a}\cdot A\cdot v{\left(t\right)}^{2}}{2m}-g\left(\rho_{r}\cdot \cos\theta +\sin\theta \right)$$

where t, $r_{\mathrm{wh}}$, m, $C_{a}$, $\rho_{a}$, A, g, $\rho_{r}$ and θ show the moment, wheel radius, vehicle mass, air drag coefficient, air density, frontal area, gravitational acceleration, rolling resistance coefficient and slope angle, respectively. While the clutch is rigidly connected to the gearbox, the required torque $T_{\mathrm{req}}$ of wheels can be shown as

(3) $$T_{\mathrm{req}}(t)=T_{\mathrm{ps}}(t)\cdot i_{\mathrm{gb}}(t)\cdot i_{d}\cdot \eta_{\mathrm{ch}}$$

where $T_{\mathrm{ps}}$, $i_{\mathrm{gb}}$, $i_{d}$ and $\eta_{\mathrm{ch}}$ denote the output torque of the power source, gear ratio of gearbox, driveline reduction ratio and system mechanical transmission efficiency, respectively.

For the variables that affect v shown in (1)–(3), $i_{d}$ and $\eta_{\mathrm{ch}}$ are fixed values after the vehicle system is designed completely. Considering that environmental factors change slightly during driving, $r_{\mathrm{wh}}$, m, $C_{a}$, $\rho_{a}$, A, g and $\rho_{r}$ are also considered as fixed values in this paper. In other words, the variables that affect vehicle speed mainly include $T_{\mathrm{ps}}$, $i_{\mathrm{gb}}$, θ and a. Here, $T_{\mathrm{ps}}$ is strongly related to the pedal degree controlled by the driver, characterized by the pedal degree p. In addition, $i_{\mathrm{gb}}$ relating to gear signal is a logical variable. p, θ and a are continuous variables changing with t. Therefore, the speed prediction process can be written as

(4) $$\hat{v}(t+j)=\left\{\begin{array}{l} f_{i_{\mathrm{gb}}(t+j-1)}\left(v(t),p(t),\theta (t),a(t)\right),j=1\\ f_{i_{\mathrm{gb}}(t+j-1)}\left(\hat{v}(t+j-1),\hat{p}(t+j-1),\hat{\theta }(t+j-1),\hat{a}(t+j-1)\right),j\geq 2\;\;\;\mathrm{and}\;\;\;j\in N+\\ i_{\mathrm{gb}}(t+j-1)\in [1,2,3,\ldots ,n] \end{array}\right.$$

where $\hat{v}$, $\hat{p}$, $\hat{\theta }$ and $\hat{a}$ are the predictive variables. j and n are the prediction horizon size and maximum gear of gearbox, respectively. Namely, the essence of VSP is to determine function $f_{i_{\mathrm{gb}}(t+j-1)}$.

For clarifying the relationship between v and p, θ and a under different gears, we take an 80-ton mining truck as the research object, to analyze the impact degree of p, θ and a on v under off-road conditions in the next section.

### 3.2. Working Condition Data Collection and Analysis

**Figure 2:**
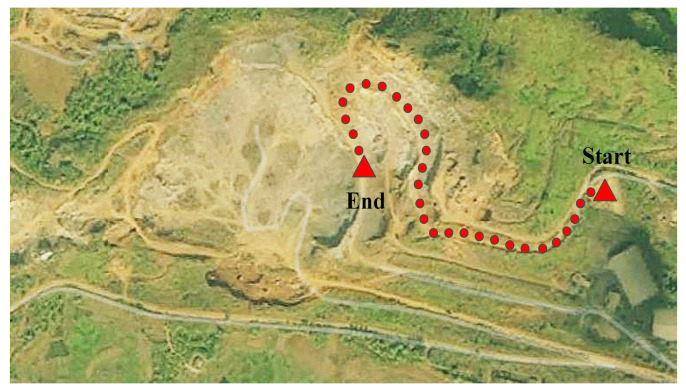
Overview of the cement mine.

**Figure 3:**
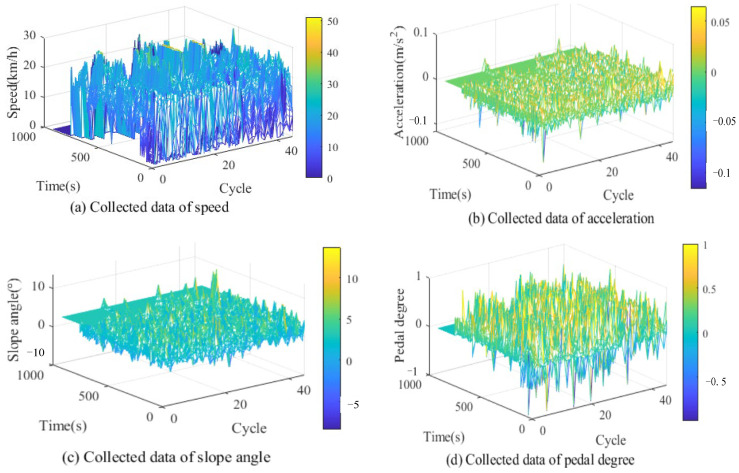
Collected working condition data.

We chose the cement mine located in Fujian Province as the operating scenario, as shown in Figure 2. The single cycle distance is about 3 km. The working condition data of an 80-ton mining truck with a five-speed gearbox was collected continuously for 30 days, and the specific data information of p, θ, a and v are shown in Figure 3.

As shown in Figure 3, in view of the nonlinearity of the collected parameters, the Spearman data statistics tool [36] is employed to analyze the correlation between parameters in this paper. The Spearman tool uses the following equation to analyze the impact degree of multiple parameters on a single parameter, for providing a basis to fit the nonlinear relationship between parameters.

(5) $$r_{s}=1-\frac{6\sum_{i=1}^{M}d_{i}^{2}}{M(M^{2}-1)},r_{s}\in [-1,1]$$

where $r_{s}$ shows the related coefficient of two parameters. $d_{i}$ represents the rank difference of two parameters. M denotes the number of samples. According to the principle of Spearman, $r_{s}=0$, $r_{s}>0$ and $r_{s}<0$ indicate that there is no correlation, positive correlation and negative correlation between the two parameters, respectively.

**Figure 4:**
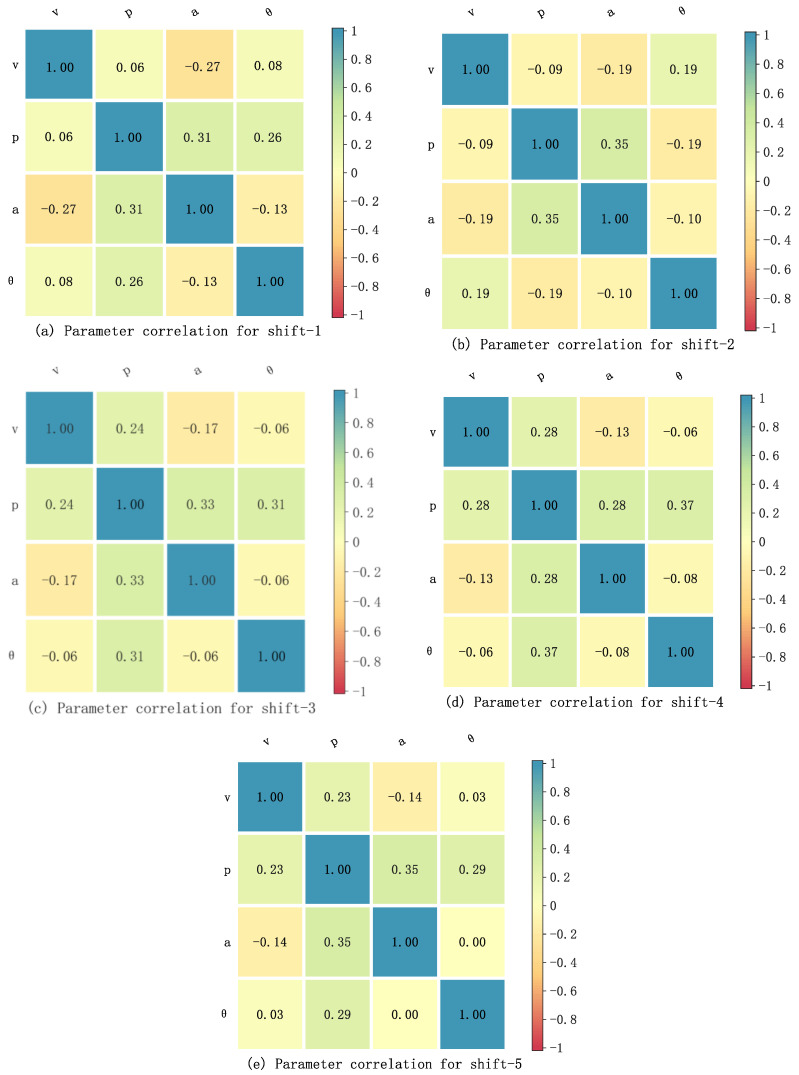
Correlation analysis between parameters.

After calculation, the relationship between each mentioned parameter (i.e., p, θ and a) and v under different gears are shown in Figure 4. It is thus clear that the related coefficients of p, θ and a on v are not 0, showing that the influence of p, θ and a on v is obvious. As mentioned above, with the help of the fitting ability of NNs to nonlinear systems, a two-layer speed prediction framework based on BPNN-LSTM is proposed in the next section.

## 4. Vehicle Speed Prediction Based on BPNN-LSTM

In this section, the double-layer VSP framework is presented, which includes speed prediction and information update layers. The speed prediction layer established by using LSTM is to predict vehicle speed in the horizon, and the information update layer built using a BPNN is to update the prediction information.

### 4.1. BPNN Prediction Models of $\hat{p}$ and $\hat{\theta }$

**Figure 5:**
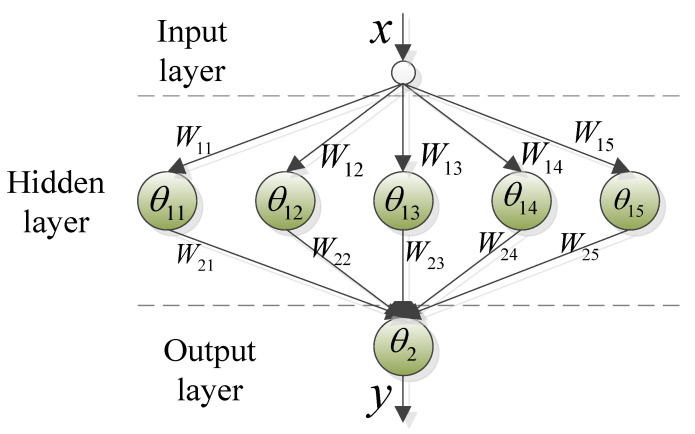
BPNN structure.

As known from (4), for achieving speed prediction in the prediction horizon, $\hat{p}$, $\hat{\theta }$ and $\hat{a}$ need to be obtained in advance. Here, once the vehicle speeds at adjacent times are known, $\hat{a}$ can be determined according to (1). For $\hat{p}$ and $\hat{\theta }$, although they can be considered as changing over time, their change process is not affected by short-term historical information. In this paper, the BPNN is employed to establish the prediction models of $\hat{p}$ and $\hat{\theta }$. According to the relationship between the number of input and output parameters, which satisfies (11) in [37], this paper selects a BPNN with 1-5-1 structure, as shown in Figure 5. The corresponding model can be described as

(6) $$\left\{\begin{array}{l} H_{\mathrm{in}}=x\times W_{1}+\theta_{1}\\ H_{\mathrm{ou}}=f\left(H_{\mathrm{in}}\right)\\ y=h(H_{\mathrm{ou}}\times W_{2}+\theta_{2}) \end{array}\right.$$

where x and y represent the input and output vectors of BPNN, respectively. $W_{1}$ and $W_{2}$ describe the weight vectors of the input-layer to hidden-layer and hidden-layer to output-layer, respectively. $\theta_{1}$ and $\theta_{2}$ show the threshold vectors of the hidden-layer and output-layer, respectively. f and h represent the activation functions of the hidden-layer nodes and output-layer nodes [37], here selecting the tansig and purelin functions, respectively. The above variables can be further expressed as

(7) $$\left\{\begin{array}{l} x=p\mathrm{or}\;\theta \\ W_{1}=\left[W_{11},W_{12},W_{13},W_{14},W_{15}\right]\\ \theta_{1}=\left[\theta_{11},\theta_{12},\theta_{13},\theta_{14},\theta_{15}\right]\\ W_{2}={\left[W_{21},W_{22},W_{23},W_{24},W_{25}\right]}^{T}\\ \theta_{2}=\theta_{2}\\ y=\hat{p}\mathrm{or}\;\hat{\theta } \end{array}\right.$$

Then, using the collected slope angle and pedal degree data to train weights and thresholds in (6), the prediction models of $\hat{p}$ and $\hat{\theta }$ are determined.

In contrast, the change process of v possesses the following characteristics:

(1) The short-term historical speed influences current speed and future speed, i.e., information inheritance;
(2) The impact of long-term historical speed on current speed will be reduced, i.e., information forgetting.

The NNs that do not consider the continuous relationship between data hardly characterize these characteristics, while the LSTM can effectively represent this process. Thus, the VSP model based on the LSTM network is built in the next section.

### 4.2. LSTM Vehicle Speed Prediction Network

**Figure 6:**
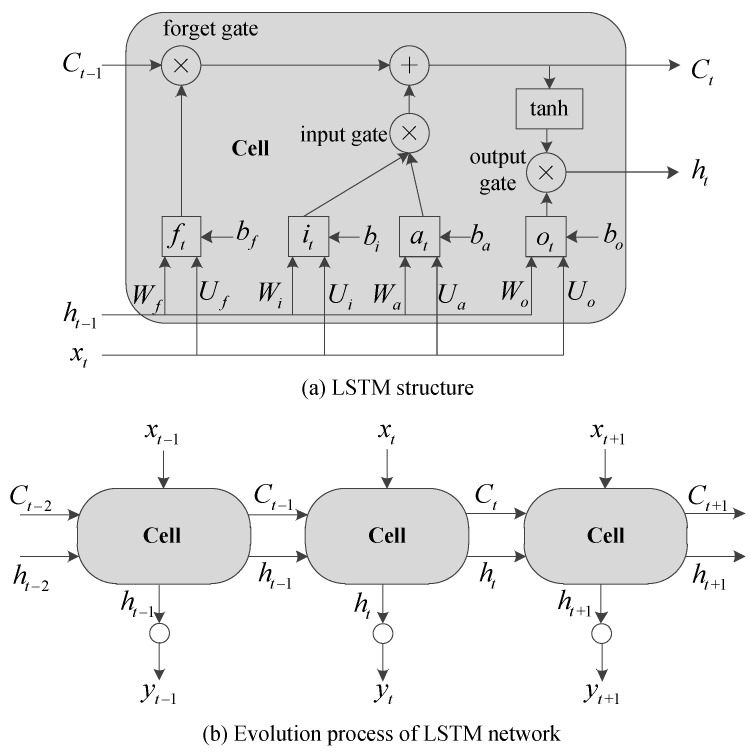
LSTM structure and network.

The framework of LSTM is shown in Figure 6a, regarded as a cell consisting of forgetting, input and output gates. Here, the forget gate controls the impact of historical information on current information. The input gate controls the current state of the cell by using the current input signal and the cell state at the last time. The output gate determines the output of the cell. The transmission relationship of these three gates is as follows:

(8) $$f_{t}=\sigma (U_{f}x_{t}+W_{f}h_{t-1}+b_{f})$$

(9) $$\left\{\begin{array}{l} i_{t}=\sigma (U_{i}x_{t}+W_{i}h_{t-1}+b_{i})\\ a_{t}=\tanh(U_{a}x_{t}+W_{a}h_{t-1}+b_{a})\\ C_{t}=C_{t-1}⊙f_{t}+i_{t}⊙a_{t} \end{array}\right.$$

(10) $$\left\{\begin{array}{l} o_{t}=\sigma (U_{o}x_{t}+W_{o}h_{t-1}+b_{o})\\ h_{t}=o_{t}⊙\tanh(C_{t}) \end{array}\right.$$

where $x_{t}$ denotes the input variable matrix at time t. σ expresses the activation function, replaced by a Sigmoid function in this paper. Both U and W with subscript represent the weight matrix. b with subscript shows the bias matrix. $C_{t}$ and $h_{t}$ are the cell state and state output at time t, respectively. $f_{t}$ indicates the memory degree of $C_{t-1}$ at time t. ⊙ denotes the Hadamard product. When U, W, b, $C_{t-1}$, $h_{t-1}$ and $x_{t}$ are defined, $h_{t}$ can be calculated. According to Figure 6b, the output $y_{t}$ of the LSTM network can be obtained by

(11) $$y_{t}=\sigma \left(Vh_{t}+c\right)$$

where V and c indicate the weight and bias matrixes of network output node, respectively.

**Figure 7:**
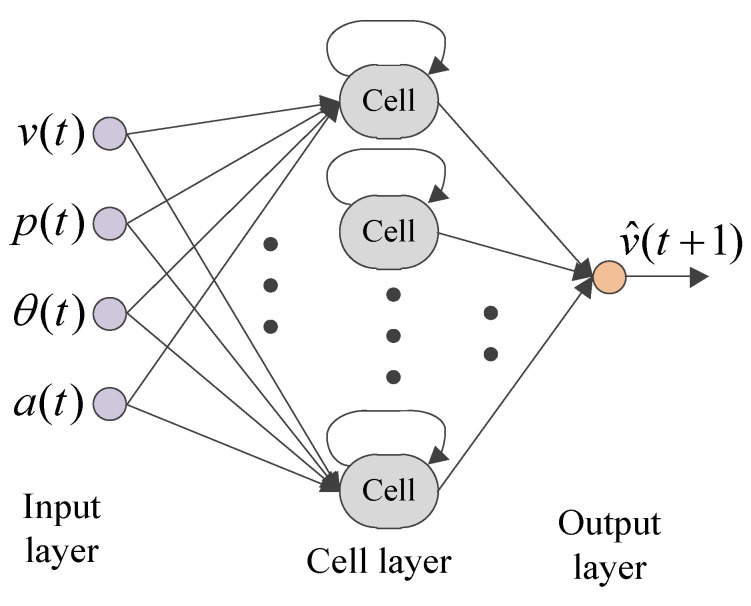
The VSP network based on LSTM.

It can be seen from (8)–(10) that the LSTM network state information at the previous moment is inherited by the network outputs at the next moment. This inheritance relationship can effectively characterize the speed prediction model evolved by time series. Therefore, taking the speed, pedal degree, slope angle, and acceleration as inputs and the predicted speed as outputs, this paper establishes a VSP model by means of an LSTM network. Specifically, the VSP network based on LSTM is shown in Figure 7. Here, $x_{t}$ in (8)–(10) and $y_{t}$ in (11) can be further expressed as $x_{t}={\left[v(t),p(t),\theta (t),a(t)\right]}^{T}$ and $y_{t}=\hat{v}(t+1)$, $\hat{v}$ represents the predicted vehicle speed.

Obviously, once U, V, W, b and c of the LSTM network are determined, we are able to use (8)–(11) to realize the speed prediction online. Furthermore, for ensuring the prediction accuracy of the LSTM network, the gradient descent method is used to train and update U, V, W, b and c until the prediction error L meets the target value:

(12) $$\left\{\begin{array}{l} L=\frac{1}{2}\sum_{t=1}^{\tau }{\left\|L_{t}\right\|}^{2}\leq \varepsilon \\ L_{t}=y_{t}-{\hat{y}}_{t} \end{array}\right.$$

where ${\hat{y}}_{t}$ is the real value of $y_{t}$, and τ is the training data sequence size. ε is the target error. To facilitate analysis, L is divided into two parts: error ${\overset{\leftarrow }{L}}_{t}$ from time 1 to time t and error ${\vec{L}}_{t}$ from time t+1 to time τ, namely,

(13) $$L=\left\{\begin{array}{l} {\overset{\leftarrow }{L}}_{t}+{\vec{L}}_{t},t<\tau \\ {\overset{\leftarrow }{L}}_{t},t=\tau \end{array}\right.$$

Then, the parameter training and learning process is analyzed. Firstly, considering the information inheritance of the LSTM, $C_{t}$ and $h_{t}$ are derived by L, shown as

(14) $$\left\{\begin{array}{c} \left\{\begin{array}{l} \delta_{h}^{t}=\frac{\partial L}{\partial h_{t}}=\frac{\partial {\overset{\leftarrow }{L}}_{\tau }}{\partial y_{\tau }}\frac{\partial y_{\tau }}{\partial h_{\tau }}=\left|y_{\tau }-{\hat{y}}_{\tau }\right|\dot{\sigma }\left(Vh_{\tau }+c\right)V\\ \delta_{C}^{t}=\frac{\partial L}{\partial C_{t}}=\frac{\partial {\overset{\leftarrow }{L}}_{\tau }}{\partial y_{\tau }}\frac{\partial y_{\tau }}{\partial h_{\tau }}\frac{\partial h_{\tau }}{\partial C_{\tau }}=\delta_{h}^{\tau }⊙o_{\tau }⊙\left(1-\tanh^{2}(C_{\tau })\right) \end{array}\right.,t=\tau \\ \left\{\begin{array}{l} \delta_{h}^{t}=\frac{\partial L}{\partial h_{t}}=\frac{\partial {\overset{\leftarrow }{L}}_{t}}{\partial y_{t}}\frac{\partial y_{t}}{\partial h_{t}}+\frac{\partial {\vec{L}}_{t}}{\partial h_{t+1}}\frac{\partial h_{t+1}}{\partial h_{t}}=\left|y_{t}-{\hat{y}}_{t}\right|\dot{\sigma }\left(Vh_{t}+c\right)V+\frac{\partial h_{t+1}}{\partial h_{t}}\delta_{h}^{t+1}\\ \delta_{C}^{t}=\frac{\partial L}{\partial C_{t}}=\frac{\partial {\overset{\leftarrow }{L}}_{t}}{\partial y_{t}}\frac{\partial y_{t}}{\partial h_{t}}\frac{\partial h_{t}}{\partial C_{t}}+\frac{\partial {\vec{L}}_{t}}{\partial C_{t+1}}\frac{\partial C_{t+1}}{\partial C_{t}}=\delta_{h}^{t}⊙o_{t}⊙\left(1-\tanh^{2}(C_{t})\right)+\delta_{C}^{t+1}⊙f_{t+1} \end{array}\right.,t<\tau \end{array}\right.$$

Because σ is the Sigmoid function in this paper, $\frac{\partial h_{t+1}}{\partial h_{t}}$ can be further expressed as

(15) $$\frac{\partial h_{t+1}}{\partial h_{t}}=\frac{\partial \left[o_{t+1}⊙\tanh(C_{t+1})\right]}{\partial h_{t}}=\frac{\partial o_{t+1}}{\partial h_{t}}⊙\tanh(C_{t+1})+o_{t+1}⊙\frac{\partial \tanh(C_{t+1})}{\partial h_{t}}$$

Herein,

(16) $$\left\{\begin{array}{l} \frac{\partial o_{t+1}}{\partial h_{t}}=o_{t+1}⊙(1-o_{t+1})W_{o}\\ \frac{\partial \tanh(C_{t+1})}{\partial h_{t}}=\left[1-\tanh^{2}(C_{t+1})\right]\frac{\partial C_{t+1}}{\partial h_{t}} \end{array}\right.$$

(17) $$\begin{array}{ll} \frac{\partial C_{t+1}}{\partial h_{t}} & =\frac{\partial \left[C_{t}⊙f_{t+1}+i_{t+1}⊙a_{t+1}\right]}{\partial h_{t}}=\frac{\partial \left[C_{t}⊙f_{t+1}\right]}{\partial h_{t}}+\frac{\partial \left[i_{t+1}⊙a_{t+1}\right]}{\partial h_{t}}\\ & =\frac{\partial \left[C_{t}⊙f_{t+1}\right]}{\partial h_{t}}+\frac{\partial i_{t+1}}{\partial h_{t}}⊙a_{t+1}+i_{t+1}⊙\frac{\partial a_{t+1}}{\partial h_{t}} \end{array}$$

Furthermore,

(18) $$\frac{\partial \left[C_{t}⊙f_{t+1}\right]}{\partial h_{t}}=C_{t}⊙f_{t+1}⊙\left(1-f_{t+1}\right)W_{f}$$

(19) $$\frac{\partial i_{t+1}}{\partial h_{t}}⊙a_{t+1}=i_{t+1}⊙\left(1-i_{t+1}\right)⊙a_{t+1}W_{i}$$

(20) $$i_{t+1}⊙\frac{\partial a_{t+1}}{\partial h_{t}}=i_{t+1}⊙\left(1-a_{t+1}^{2}\right)W_{a}$$

We let $\Delta C=o_{t+1}⊙\left[1-\tanh^{2}(C_{t+1})\right]$, (15) can be written as

(21) $$\begin{array}{ll} \frac{\partial h_{t+1}}{\partial h_{t}} & =o_{t+1}⊙(1-o_{t+1})⊙\tanh(C_{t+1})W_{o}+\Delta C⊙C_{t}⊙f_{t+1}⊙\left(1-f_{t+1}\right)W_{f}+\ldots \\ & \Delta C⊙i_{t+1}⊙\left(1-i_{t+1}\right)⊙a_{t+1}W_{i}+\Delta C⊙i_{t+1}⊙\left(1-a_{t+1}^{2}\right)W_{a} \end{array}$$

Then, the gradient variations of $W_{f}$, $U_{f}$, $b_{f}$, $W_{a}$, $U_{a}$, $b_{a}$, $W_{i}$, $U_{i}$, $b_{i}$, $W_{o}$, $U_{o}$, $b_{o}$, V and c can be shown as

(22) $$\left\{\begin{array}{l} \frac{\partial L}{\partial W_{f}}=\sum_{t=1}^{\tau }\delta_{C}^{t}⊙C_{t-1}⊙f_{t}⊙\left(1-f_{t}\right)h_{t-1}^{T}\\ \frac{\partial L}{\partial U_{f}}=\sum_{t=1}^{\tau }\delta_{C}^{t}⊙C_{t-1}⊙f_{t}⊙\left(1-f_{t}\right)x_{t}\\ \frac{\partial L}{\partial b_{f}}=\sum_{t=1}^{\tau }\delta_{C}^{t}⊙C_{t-1}⊙f_{t}⊙\left(1-f_{t}\right) \end{array}\right.$$

(23) $$\left\{\begin{array}{l} \frac{\partial L}{\partial W_{a}}=\sum_{t=1}^{\tau }\delta_{C}^{t}⊙i_{t}⊙\left(1-a_{t}^{2}\right)h_{t-1}^{T}\\ \frac{\partial L}{\partial U_{a}}=\sum_{t=1}^{\tau }\delta_{C}^{t}⊙i_{t}⊙\left(1-a_{t}^{2}\right)x_{t}\\ \frac{\partial L}{\partial b_{a}}=\sum_{t=1}^{\tau }\delta_{C}^{t}⊙i_{t}⊙\left(1-a_{t}^{2}\right) \end{array}\right.$$

(24) $$\left\{\begin{array}{l} \frac{\partial L}{\partial W_{i}}=\sum_{t=1}^{\tau }\delta_{C}^{t}⊙a_{t}⊙i_{t}⊙\left(1-i_{t}\right)h_{t-1}^{T}\\ \frac{\partial L}{\partial U_{i}}=\sum_{t=1}^{\tau }\delta_{C}^{t}⊙a_{t}⊙i_{t}⊙\left(1-i_{t}\right)x_{t}\\ \frac{\partial L}{\partial b_{i}}=\sum_{t=1}^{\tau }\delta_{C}^{t}⊙a_{t}⊙i_{t}⊙\left(1-i_{t}\right) \end{array}\right.$$

(25) $$\left\{\begin{array}{l} \frac{\partial L}{\partial W_{o}}=\sum_{t=1}^{\tau }\delta_{h}^{t}⊙\tanh\left(C_{t}\right)⊙o_{t}⊙\left(1-o_{t}\right)h_{t-1}^{T}\\ \frac{\partial L}{\partial U_{o}}=\sum_{t=1}^{\tau }\delta_{h}^{t}⊙\tanh\left(C_{t}\right)⊙o_{t}⊙\left(1-o_{t}\right)x_{t}\\ \frac{\partial L}{\partial b_{o}}=\sum_{t=1}^{\tau }\delta_{h}^{t}⊙\tanh\left(C_{t}\right)⊙o_{t}⊙\left(1-o_{t}\right) \end{array}\right.$$

(26) $$\left\{\begin{array}{l} \frac{\partial L}{\partial V}=\sum_{t=1}^{\tau }\left|y_{t}-{\hat{y}}_{t}\right|y_{t}\left(1-y_{t}\right)h_{t}\\ \frac{\partial L}{\partial c}=\sum_{t=1}^{\tau }\left|y_{t}-{\hat{y}}_{t}\right|y_{t}\left(1-y_{t}\right) \end{array}\right.$$

Further, $W_{f}$, $U_{f}$, $b_{f}$, $W_{a}$, $U_{a}$, $b_{a}$, $W_{i}$, $U_{i}$, $b_{i}$, $W_{o}$, $U_{o}$, $b_{o}$, V and c can be updated by using the following equation:

(27) $$\psi^{t+\tau }=\psi^{t}-\eta \cdot \frac{\partial L}{\partial \psi }$$

where ψ represents one of the forms of $W_{f}$, $U_{f}$, $b_{f}$, $W_{a}$, $U_{a}$, $b_{a}$, $W_{i}$, $U_{i}$, $b_{i}$, $W_{o}$, $U_{o}$, $b_{o}$, V and c. η∈(0,1] denotes the learning factor.

After determining the forward transmission and reverse learning process of the LSTM, the collected data can be used to train the LSTM network, and the trained network can be used for online speed prediction.

### 4.3. Double-Layer VSP Model Based on BPNN-LSTM

**Figure 8:**
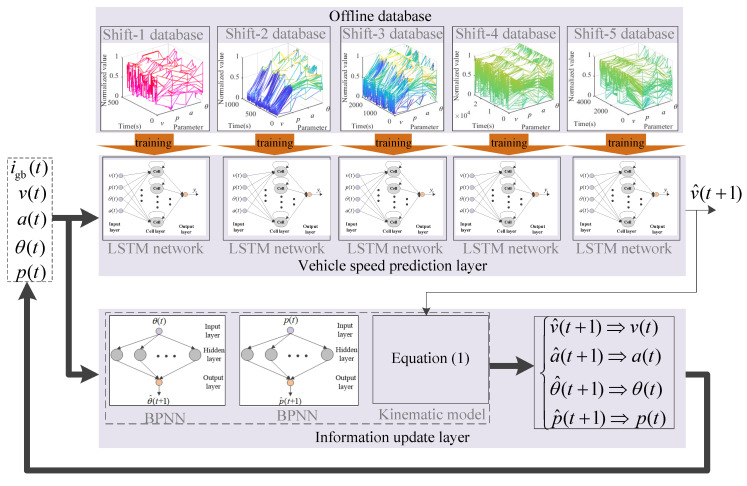
A double-layer VSP architecture based on BPNN-LSTM.

**Figure 9:**
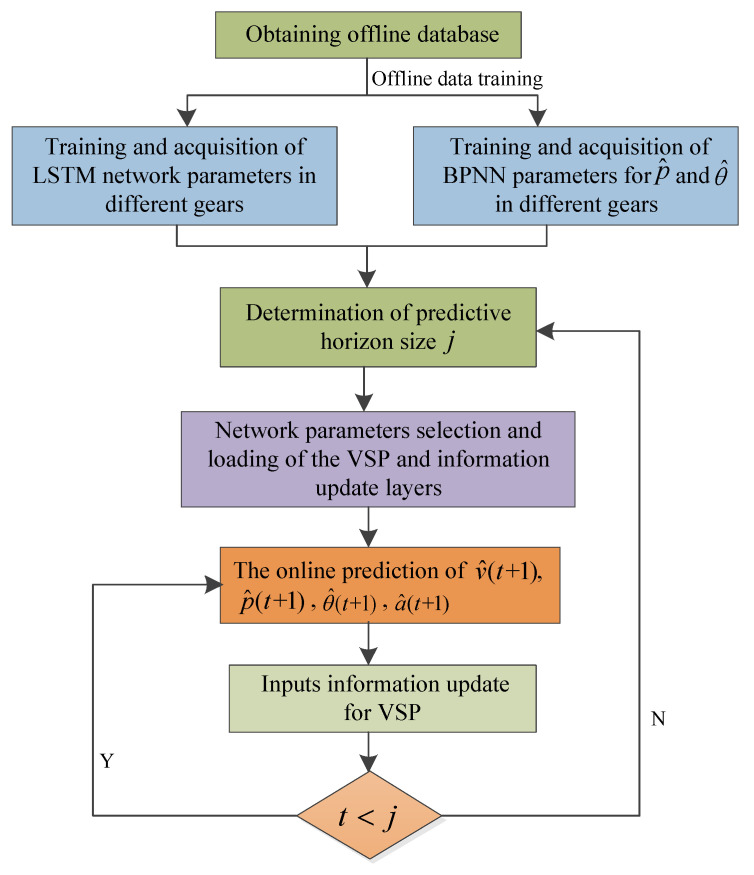
The specific flow diagram of the proposed BPNN-LSTM algorithm.

As shown in Figure 4, the influence of p, θ and a on v is different under different gears. In order to improve the VSP accuracy, in this paper, a double-layer VSP architecture based on BPNN-LSTM is presented, shown in Figure 8, including vehicle speed prediction layer and information update layer. Here, the off-line databases are used to train the LSTM network and BPNN. Firstly, according to current $i_{\mathrm{gb}}(t)$, the vehicle speed prediction layer selects the trained LSTM network corresponding to gear. Then, based on current $\hat{v}$, $\hat{p}$, $\hat{\theta }$ and $\hat{a}$, the next moment speed $\hat{v}(t+1)$ is predicted online by using the selected trained LSTM network from the VSP layer. The information update layer includes BPNN prediction models of $\hat{p}$ and $\hat{\theta }$, and the kinematic model of $\hat{a}$. Here, the next moment $\hat{p}(t+1)$ and $\hat{\theta }(t+1)$ is predicted by using the trained BPNN prediction models of $\hat{p}$ and $\hat{\theta }$. The next moment $\hat{a}$ is predicted by utilizing the equation (1). Namely, the information update layer is used to update $\hat{v}$, $\hat{p}$, $\hat{\theta }$ and $\hat{a}$. The specific flow diagram of the proposed algorithm is shown in Figure 9. Correspondingly, the specific implementation process is as follows.

Step 1: By means of Equations (12) to (27), the LSTM network utilizes the databases of different gears to train network parameters and obtain the LSTM network parameters of different gears. On the basis of Section 4.1, the BPNN uses the slope angle and pedal degree data of different gears to train their network parameters, and obtain BPNN parameters of different gears for $\hat{p}$ and $\hat{\theta }$, respectively.

Step 2: The predictive horizon size j is determined.

Step 3: According to current $i_{\mathrm{gb}}(t)$, the VSP layer selects the trained LSTM network parameters of the corresponding gear and loads them into the LSTM network. The trained BPNN parameters of the corresponding gear for $\hat{p}$ and $\hat{\theta }$ are loaded into their respective networks.

Step 4: Based on LSTM network, the VSP layer uses current v(t), p(t), θ(t) and a(t) to predict next moment speed $\hat{v}(t+1)$ online.

Step 5: By means of the BPNN prediction models of $\hat{p}$ and $\hat{\theta }$, and vehicle kinematics equation, the information update layer utilizes p(t), θ(t) and a(t) to predict $\hat{p}(t+1)$, $\hat{\theta }(t+1)$ and $\hat{a}(t+1)$, and updates the input information of the VSP layer in the next moment.

Step 6: If t<j, then back to Step 4; Otherwise, back to Step 2 until the trip is complete.

## 5. Results and Discussion

In this section, for verifying the speed prediction effect of the proposed double-layer VSP method, the analytical method, BPNN prediction method, and RNN prediction method will be simulated and compared with the double-layer VSP method under the same experimental conditions.

### 5.1. Experimental Conditions Setting

#### 5.1.1. Experimental Scenario

This paper selects the mining truck operation scenario shown in Figure 2 and a loader operation scenario for simulation.

#### 5.1.2. Performance Evaluation Methods

(1) The prediction effect of the vehicle speed prediction method is evaluated by the following errors:

(28) $$\left\{\begin{array}{l} AE=v_{i}-{\hat{v}}_{i}\\ MAE=\frac{1}{M}\sum_{i=1}^{M}\left|v_{i}-{\hat{v}}_{i}\right|\\ RMSE=\sqrt{\frac{1}{M}\sum_{i=1}^{M}{\left(v_{i}-{\hat{v}}_{i}\right)}^{2}} \end{array}\right.$$

where AE, MAE and RMSE represent the absolute, mean-absolute and root-mean-square errors, respectively. $v_{i}$ and ${\hat{v}}_{i}$ denote the real and predictive values of the vehicle speed, respectively.

(2) In order to verify the correlation between the actual speed and the predicted speed obtained by different VSP methods, the Pearson correlation coefficient is used to describe the dependence between the predicted and actual speed. The specific expression is as follows:

(29) $$R=\frac{\sum_{i=1}^{n}\left(v_{i}-\bar{v})\cdot ({\hat{v}}_{i}-\bar{\hat{v}}\right)}{\sqrt{\sum_{i=1}^{n}{\left(v_{i}-\bar{v}\right)}^{2}\sum_{i=1}^{n}{\left({\hat{v}}_{i}-\bar{\hat{v}}\right)}^{2}}}$$

where R is the Pearson correlation coefficient of $v_{i}$ and ${\hat{v}}_{i}$. $\bar{v}$ and $\bar{\hat{v}}$ show the average values of the actual and predicted speed, respectively. n is the number of data.

#### 5.1.3. Simulation Platform

**Figure 10:**
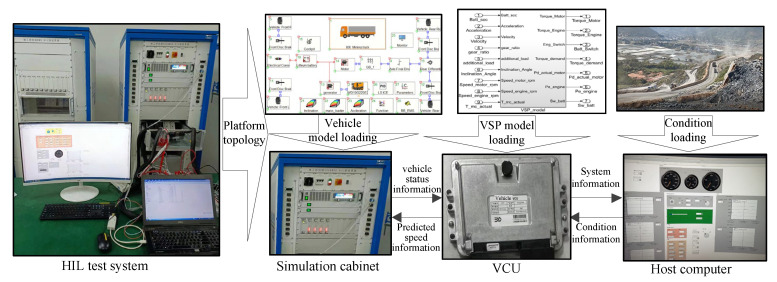
VSP simulation platform.

The simulation platform for VSP consists of an HIL bench and vehicle control unit (VCU), shown in Figure 10.

### 5.2. Vehicle Speed Prediction Analysis

#### 5.2.1. Horizon Size *j* Selection

**Figure 11:**
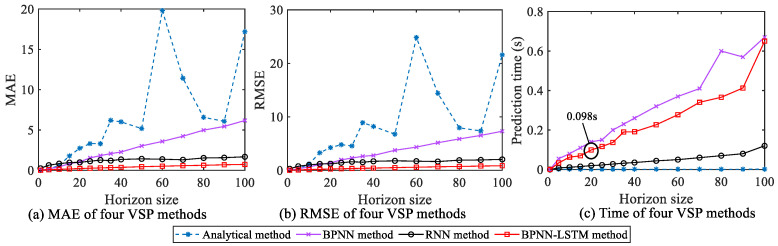
The changing relationships of MAE, RMSE and prediction time of four methods with horizon size.

According to (4), j not only characterizes the size of the prediction horizon, but also affects the online prediction accuracy of the vehicle speed. Thus, it is necessary to choose a proper j to guarantee the prediction accuracy of the vehicle speed. For analyzing the influence of different horizon sizes on VSP performance, taking the collected working condition information of the mining truck as an example, the analytical method, BPNN, RNN and the designed BPNN-LSTM prediction methods are simulated and compared. The changing relationships of MAE, RMSE and prediction time of the four methods with j are shown in Figure 11. It can be seen from Figure 11a,b that the MAE and RMSE of the BPNN-LSTM prediction method are smaller than that of the other three methods. Considering that one running time of VCU in the real vehicle is 100 ms, in order to realize the vehicle speed prediction in 100 ms, the initial value of j is set to 20 based on Figure 11c.

#### 5.2.2. VSP in Mining Truck Operation Scenario

**Figure 12:**
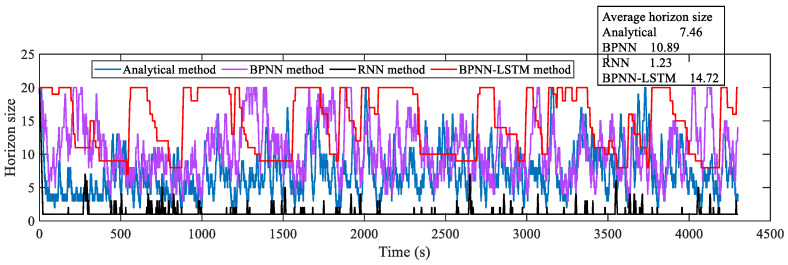
Horizon size of four VSP methods in mining truck operation scenario.

**Figure 13:**
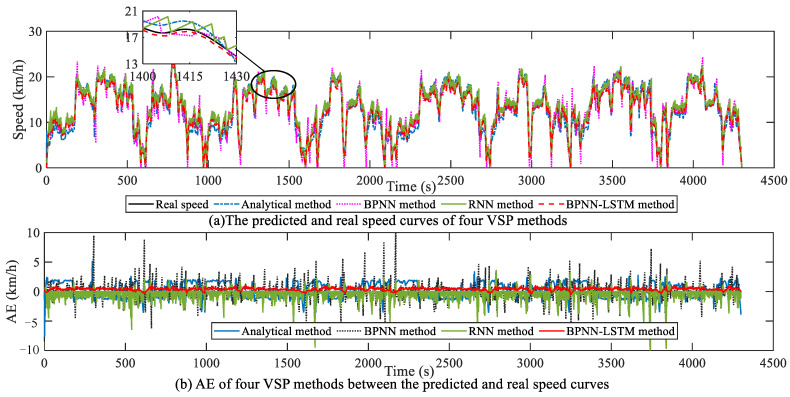
Speed prediction analysis in mining truck operation scenario.

For verifying the speed prediction effect of the proposed VSP method, on the basis of selecting five cycles of the mining truck operation scenario, the analytical method, BPNN, RNN and BPNN-LSTM VSP methods are simulated on the HIL platform. To ensure the accuracy and real-time performance of speed prediction, the absolute value range of AE is set to [0.18 km/h, 0.72 km/h], and the horizon size is dynamically adjusted according to the set AE range. Under the mining truck operation scenario, the horizon size changing curves of four VSP methods are presented in Figure 12. It is thus clear that the average horizon size of the BPNN-LSTM prediction method is larger compared with the other three methods. The relations and AE of the predicted and real speed curves for the four methods are indicated in Figure 13. Correspondingly, the MAE, RMSE and R of the four methods are given in Table 1.

First of all, according to Figure 13a, although the predicted speed trajectories of the four VSP methods can follow the real speed trajectory, their corresponding AE ranges are diverse. Specifically, under the mining truck operation scenario, the AE ranges of the analytical, BPNN, RNN and BPNN-LSTM prediction methods are [−7.56 km/h, 5.11 km/h], [−7.46 km/h, 10.57 km/h], [−11.26 km/h, 3.58 km/h] and [−0.5 km/h, 1.11 km/h], respectively. It can be seen that the AE variation range of the BPNN-LSTM method is the smallest among four VSP methods.

Then, it can be seen from Table 1 that the Pearson correlation coefficient R of the BPNN-LSTM method is closest to 1, namely, the prediction performance of the BPNN-LSTM method is better. In addition, it is thus clear from Table 1 that the MAE and RMSE of the designed BPNN-LSTM methods are smaller than those of the other three prediction methods. Specifically, compared with the analytical method, the BPNN, RNN, MAE and RMSE of the BPNN-LSTM prediction method can reduce by 63.83% and 64.78%, 39.83% and 61.49%, 34.36% and 56.72%, respectively.

#### 5.2.3. VSP in Loader Operation Scenario

**Figure 14:**
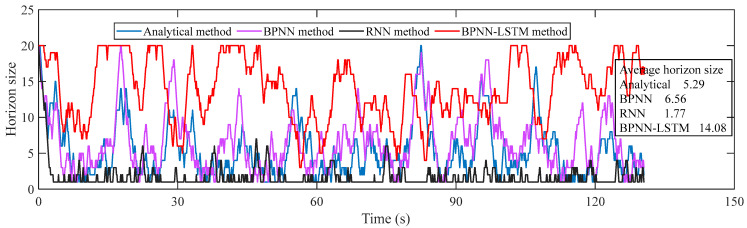
Horizon size of four VSP methods in loader operation scenario.

**Figure 15:**
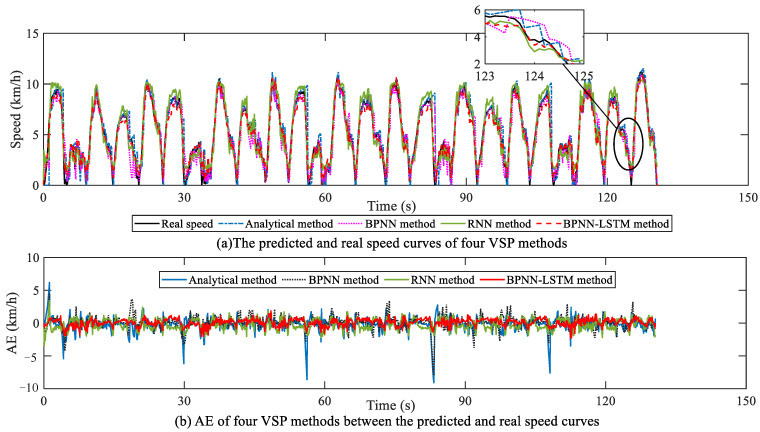
Speed prediction analysis in loader operation scenario.

To further verify the universality of the proposed BPNN-LSTM prediction method for working conditions, the analytical, BPNN, RNN and BPNN-LSTM VSP methods are also compared in a loader operation scenario, selecting five cycles to simulate on the HIL platform. The horizon size of the four VSP methods is also dynamically adjusted according to the set AE range in Section 5.2.2, as described in Figure 14. Obviously, in a loader operation scenario, the average horizon size of the BPNN-LSTM prediction method is also the largest among the four methods. The relations and AE of the predicted and real speed trajectories for four methods are described in Figure 15 under the loader operation scenario. Accordingly, the MAE, RMSE and R of the four methods are presented in Table 2.

Firstly, based on Figure 15 and Table 2, contrasted with the analytical, BPNN and RNN prediction methods, the AE changing range of the speed trajectory predicted by the BPNN-LSTM method can reduce in different degrees, and the corresponding MAE and RMSE can also decrease in various degrees. Specifically, under the loader operation scenario, the AE ranges of the analytical method, and the BPNN, RNN and BPNN-LSTM VSP methods are [−9.08 km/h, 6.19 km/h], [−7.95 km/h, 4.54 km/h], [−3.45 km/h, 3.43 km/h] and [−2.25 km/h, 1.87 km/h], respectively. Secondly, compared with the other three prediction methods, it is thus clear from Table 2 that the Pearson correlation coefficient R of the BPNN-LSTM method is also closest to 1 in the loader operation scenario. Furthermore, the MAE and RMSE of the proposed BPNN-LSTM method are less than that of the other three VSP methods. Specifically, in comparison with the analytical method, and the BPNN and RNN methods, the BPNN-LSTM prediction method can improve the precision of the MAE and RMSE by 32.45% and 50.14%, 31.81% and 40.68%, and 25.81% and 30.62%, respectively.

Based on the above analysis, whether it is in the mining truck operation scenario or the loader operation scenario, compared with the other three prediction methods, the AE changing range of the BPNN-LSTM prediction method is smaller, whose R is closest to 1. Furthermore, the MAE and RMSE of the BPNN-LSTM prediction method are also the smallest among the four prediction methods. In other words, the predicted vehicle speed trajectory by means of the BPNN-LSTM method is closer to the real vehicle speed trajectory. Correspondingly, on the premise of ensuring prediction accuracy, the average horizon size of the BPNN-LSTM prediction method under the two operation scenarios is larger than that of the other three methods. Therefore, the VSP method proposed in this paper can realize the online prediction of vehicle speed with higher prediction accuracy, and provide more valuable information for an energy management strategy.

## 6. Conclusions

In this paper, a double-layer VSP method based on BPNN-LSTM for off-road vehicles was proposed. Firstly, the VSP problem was established and the relationship between the variables in the problem was carefully analyzed. Then, the double-layer VSP framework based on BPNN-LSTM was presented, including speed prediction and information update layers. The speed prediction layer was established by using LSTM to predict vehicle speed in the horizon. Furthermore, the information update layer was built using a BPNN to update the prediction information. Finally, with the help of mining truck and loader operation scenarios, an experimental study was conducted for the proposed VSP method on the HIL platform.

Based on the analysis of the experimental results, on the premise of assuring the real-time prediction performance, the proposed VSP method can realize the prediction accuracy of the average prediction error for two operation scenarios less than 0.456 km/h, which is obviously better than the analytical, BPNN and RNN prediction methods. Correspondingly, under the premise of ensuring the VSP precision, the horizon size of the BPNN-LSTM prediction method is the largest among four prediction methods under two operation scenarios, which can provide more valuable information for optimizing energy consumption. It further improves the speed prediction ability of the NN in random road environments and provides a new solution for speed prediction of non-road vehicles.

Furthermore, our work still needs ongoing development. Firstly, the proposed BPNN-LSTM prediction method will be verified on a real vehicle. Then, we will design a reasonable energy management strategy on the basis of the verified results, giving full play to the value of speed prediction in the design of an energy management strategy for non-road vehicles.

## Figures and Tables

**Table 1:** MAE, RMSE and R of the four VSP methods in mining truck operation scenario.

Cycle	Prediction Methods	MAE	RMSE	R
Mining truck operation scenario	Analytical method	1.1600	1.3060	0.9801
	BPNN	0.6974	1.1945	0.9712
	RNN	0.6392	1.0629	0.9725
	BPNN-LSTM	0.4196	0.4600	0.9998

**Table 2:** MAE, RMSE and R of the four VSP methods in loader operation scenario.

Cycle	Prediction Methods	MAE	RMSE	R
Loader operation scenario	Analytical method	0.6748	1.1362	0.9308
	BPNN	0.6684	0.9550	0.9463
	RNN	0.6144	0.8165	0.9652
	BPNN-LSTM	0.4558	0.5665	0.9857

## Data Availability

The data presented in this study are available on request from the corresponding author. The data are not publicly available because the supporting project has a confidentiality agreement.

## Abbreviations

VSP	vehicle speed prediction	
LSTM	long short-term memory	
BPNN	backpropagation neural network	
RNN	recurrent neural network	
ITS	intelligent transportation system	
NN	neural network	
MPTM	Markov probability transfer matrix	
CNN	convolutional neural network	
GRU	gated recurrent unit	
HIL	hardware in loop	
IOT	Internet of Things	
AEKF	adaptive extended Kalman filter	
EKF	extended Kalman filter	
MSE	mean square error	
MAE	mean absolute error	
RMSE	root-mean-square error	
Oriented-HSMM	Oriented Hidden Semi-Markov Model	
SWTS	sliding window time series	
E-LLM	evolutionary least learning machine	
HMM	Hidden Markov model	
DNN	deep NN	
NIGA-SVM	niche immune genetic algorithm-support vector machine	
GA-SVM	genetic algorithm-support vector machine	
TCNs	temporal convolutional networks	
GCN	graph convolution network	
GA	genetic algorithm	
APSO-LSSVM	adaptive particle swarm optimization–least squares support vector machine	
MAPE	mean absolute percentage error	
MLPs-NN	multi-layer perception NN	
VCU	vehicle control unit	
**Variables and its unit**		
Variables	Name	Unit
l	driving distance	m
v	vehicle speed	m/s
a	acceleration	m/s^2^
t	the moment	s
$r_{wh}$	wheel radius	m
m	vehicle mass	kg
$C_{a}$	air drag coefficient	–
$\rho_{a}$	air density	kg/m^3^
A	frontal area	m^2^
g	gravitational acceleration	m/s^2^
$\rho_{r}$	rolling resistance coefficient	–
θ	slope angle	°
$T_{\mathrm{req}}$	required torque of wheels	Nm
$T_{\mathrm{ps}}$	output torque of power source	Nm
$i_{\mathrm{gb}}$	gear ratio of gearbox	–
$i_{d}$	driveline reduction ratio	–
$\eta_{\mathrm{ch}}$	system mechanical transmission efficiency	–
p	pedal degree	–
j	prediction horizon size	–
n	maximum gear of gearbox	–
